# Zebra: Static and Dynamic Genome Cover Thresholds with Overlapping References

**DOI:** 10.1128/msystems.00758-22

**Published:** 2022-09-08

**Authors:** Daniel Hakim, Stephen Wandro, Karsten Zengler, Livia S. Zaramela, Brent Nowinski, Austin Swafford, Qiyun Zhu, Se Jin Song, Antonio Gonzalez, Daniel McDonald, Rob Knight

**Affiliations:** a Department of Pediatrics, School of Medicine, University of California, San Diego, La Jolla, California, USA; b Bioinformatics and Systems Biology Program, University of California, San Diego, La Jolla, California, USA; c Department of Computer Science and Engineering, University of California, San Diego, La Jolla, California, USA; d Department of Bioengineering, University of California, San Diego, La Jolla, California, USA; e Center for Microbiome Innovation, Jacobs School of Engineering, University of California, San Diego, La Jolla, California, USA; f School of Life Sciences, Arizona State University, Tempe, Arizona, USA; g Biodesign Center for Fundamental and Applied Microbiomics, Arizona State University, Tempe, Arizona, USA; Mayo Clinic

**Keywords:** metagenomics, microbiome, read filtering

## Abstract

Assigning taxonomy remains a challenging topic in microbiome studies, due largely to ambiguity of reads which overlap multiple reference genomes. With the Web of Life (WoL) reference database hosting 10,575 reference genomes and growing, the percentage of ambiguous reads will only increase. The resulting artifacts create both the illusion of co-occurrence and a long tail end of extraneous reference hits that confound interpretation. We introduce genome cover, the fraction of reference genome overlapped by reads, to distinguish these artifacts. We show how to dynamically predict genome cover by read count and examine our model in Staphylococcus aureus monoculture. Our modeling cleanly separates both S. aureus and true contaminants from the false artifacts of reference overlap. We next introduce saturated genome cover, the true fraction of a reference genome overlapped by sample contents. Genome cover may not saturate for low abundance or low prevalence bacteria. We assuage this worry with examination of a large human fecal data set. By compositing the metric across like samples, genome cover saturates even for rare species. We note that it is a threshold on saturated genome cover, not genome cover itself, which indicates a spurious reference hit or distant relative. We present Zebra, a method to compute and threshold the genome cover metric across like samples, a recurrence to estimate genome cover and confirm saturation, and provide guidance for choosing cover thresholds in real world scenarios. Standalone genome cover and integration into Woltka are available: https://github.com/biocore/zebra_filter, https://github.com/qiyunzhu/woltka.

**IMPORTANCE** Taxonomic assignment, assigning sequences to specific taxonomic units, is a crucial processing step in microbiome analyses. Issues in taxonomic assignment affect interpretation of what microbes are present in each sample and may be associated with specific environmental or clinical conditions. Assigning importance to a particular taxon relies strongly on independence of assigned counts. The false inclusion of thousands of correlated taxa makes interpretation ambiguous, leading to underconstrained results which cannot be reproduced. The importance sometimes attached to implausible artifacts such as anthrax or bubonic plague is especially problematic. We show that the Zebra filter retrieves only the nearest relatives of sample contents enabling more reproducible and biologically plausible interpretation of metagenomic data.

The reference overlap problem in taxonomic assignment leads to ambiguously aligned reads. These ambiguities result in either assignment to a higher taxonomic rank leading to a loss in specificity, or equal distribution across all alignments resulting in assignment to extraneous species and an illusion of co-occurrence between related species (1).

KrakenUniq (2) introduces techniques to estimate and filter by unique *k*-mer count while SLIMM (3) uses preprocessing to filter by read distribution across multiple bins. However, neither tool makes use of information across samples to inform selection. Because ambiguous reads are necessarily restricted to regions where references overlap, we may use the uniformity of assigned reads to filter artifacts within and across samples overcoming the limitations of these tools to retain rare microbes that may be of phenotypic importance. We report a metric that can be composited across like samples to enable use for rare microbes and a corresponding threshold that scales dynamically by read count and reference length.

We introduce the genome cover metric, the fraction of a reference genome covered by one or more reads, as a measure of read uniformity. With sufficient reads, this metric saturates as the fraction of reference genome overlapped by sample contents. The consequence of the reference overlap problem is that an abundant species may assign read counts to both near and distant relatives within the reference, but only those closest relatives will show a high percentage of saturated genome cover. We propose a genome cover filter to remove extraneous assignments in the Web of Life (WoL) (4) from human samples.

We begin with an evaluation of monocultures where we expect taxonomic assignment to corroborate a single organism. Reads are sourced from 192 Staphylococcus aureus monoculture samples selected from lesion- and nonlesion tissues of the skin of human subjects suffering from atopic dermatitis. These data are available through the European Bioinformatics Institute (EBI) (https://www.ebi.ac.uk/ena) under the study identifier PRJEB52498 (ERP137223) and on Qiita (5): https://qiita.ucsd.edu/study/description/11919.

The Web of Life Toolkit App (Woltka [6]) accumulates read counts by splitting evenly across up to 16 matched references. Processing S. aureus monoculture in Woltka results in sporadic assignment to over 1,700 reference genomes. Table 1 displays the top 10 assignments ordered by genome cover. As expected, S. aureus MS4 dominates these samples by cover and assigned read count. Reference hits are generally filtered via relative abundance thresholds, the fraction of per-sample reads, e.g., 0.01%, and/or prevalence thresholds, the fraction of samples where the organism is detected, e.g., 10% as benchmarked for standard pipelines (7–9). Table 1 and Fig. 1 show these thresholds are insufficient to filter relatives of S. aureus.

**FIG 1:**
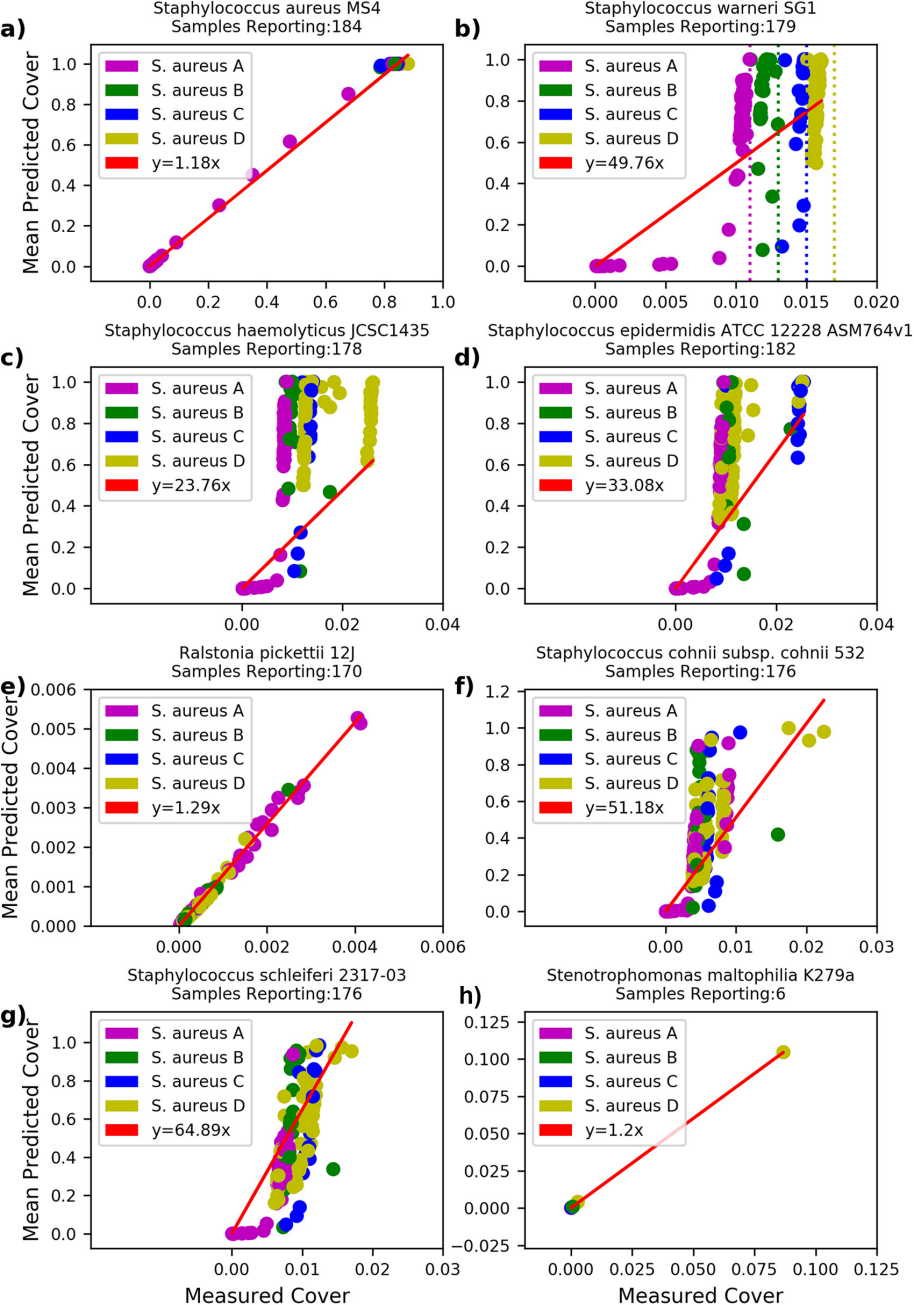
Modeling genome cover by read count differentiates low abundance contaminants from overlapping references in S. aureus monocultures. Clusters (A) to (D) determined by thresholding of Staphylococcus warneri SG1. Red indicates the line of best fit. A reported slope of 1 with no residual would indicate a perfect model fit. Mean predicted cover calculated using assigned read count and genome length assuming fixed 150 bp read length. As the number of reads increases, measured cover asymptotically approaches the overlap between true sample content and the assigned reference genome.

**TABLE 1:** Ten highest genome cover reference genomes identified in S. aureus monocultures

OGU	Covered length	Genome length	Genome cover	Predicted genome cover	Strain	Mean depth (whole-genome)	Mean depth (covered regions)	Reads	Prevalence
G001456215	2,538,281	2,709,797	93.7%	100%	Staphylococcus aureus MS4	16,194.45	17,288.73	2.9E + 08	98%
G000072485	437,397	4,851,126	9.0%	10.9%	Stenotrophomonas maltophilia K279a	0.12	1.28	3.7E + 03	4%
G000020205	404,791	5,325,729	7.6%	9.5%	Ralstonia pickettii 12J	0.10	1.33	3.6E + 03	92%
G000009865	117,070	2,697,861	4.3%	100%	Staphylococcus haemolyticus JCSC1435	373.00	8,595.77	6.7E + 06	93%
G000007645	86,798	2,564,615	3.4%	100%	Staphylococcus epidermidis ATCC 12228 ASM764v1	271.25	8,014.52	4.6E + 06	93%
G000972575	75,837	2,826,849	2.7%	100%	Staphylococcus cohnii subsp. cohnii 532	118.91	4,432.58	2.2E + 06	91%
G000332735	65,933	2,560,716	2.6%	100%	Staphylococcus warneri SG1	353.99	13,748.45	6.0E + 06	94%
G001188915	60,333	2,582,931	2.3%	100%	Staphylococcus schleiferi 2317-03	134.30	5,749.64	2.3E + 06	91%
G001471555	59,139	2,602,401	2.3%	100%	Staphylococcus capitis FDAARGOS_173	179.92	7,917.26	3.1E + 06	93%
G001068545	58,172	2,531,263	2.3%	100%	Staphylococcus epidermidis 1056_SEPI	138.31	6,018.25	2.3E + 06	93%

Nearly 7 million reads were assigned to Staphylococcus haemolyticus JCSC1435. These *S. haemolyticus* matches were identified in >90% of samples and far exceed typical filtering thresholds. These assignments are the direct result of the reference overlap problem generating the illusion of co-occurrence of multiple Staphylococcus spp. within these samples. Whereas the ~300 million reads of S. aureus overlap 94% of its genome, those assigned to *S. haemolyticus* resolve to only 4% of the *S. haemolyticus* genome with high (>8000) mean depth. The high depth regions represent the overlap between the S. aureus and *S. haemolyticus* genomes. Analogous arguments may be made for the other Staphylococcus spp. in Table 1. The reference overlap problem generates artifacts restricted to overlapping regions. We exploit this to formulate a novel filtering approach.

Fig. S1 models the genome cover metric for a given number of reads of a reference species by assuming reads are uniformly distributed and discretized to nonoverlapping buckets. Closed form mean and standard deviation for the number of covered buckets are given in Equations 3 and 4 (10). In monoculture, this model allows us to filter references whose measured cover lies outside the predicted cover range.

10.1128/msystems.00758-22.1 Figure S1 Dynamic model of genome cover parameterized by read count and genome length. Download FIG S1, TIF file, 3.6 MB. Copyright © 2022 Hakim et al. 2022 Hakim et al. https://creativecommons.org/licenses/by/4.0/ This content is distributed under the terms of the Creative Commons Attribution 4.0 International license.

There is a striking difference between the model performance on S. aureus versus other Staphylococcus species (Fig. 1). For more distant relatives, predicted genome cover is 20 to 60× higher than is measured, strongly indicating that reads are not uniformly distributed across these reference genomes. In contrast, comparing predicted genome cover against measured genome cover on a sample by sample basis based on this metric shows that for S. aureus, Stenotrophomonas maltophilia, and Ralstonia pickettii the predicted and measured cover are linearly related to within a constant factor of ~1.2 (Fig. 1). This constant factor may result from PCR-derived duplicate reads, deviation between the reference genome and the contents of the sample, and/or nonuniformity of read sampling. Thus, Ralstonia pickettii, whose cover is low but in agreement with the number of assigned reads, should be considered contamination rather than reference overlap.

Interpreting the x-intercepts as the saturating genome cover between S. aureus strains and these references, we observed vertical clusters within the Staphylococcus relatives (Fig. 1b) that indicates saturation with even a single high abundance sample. We use this fact to bound thresholds applicable to samples of unknown composition where, due to the possibility of co-occurrence, the assumptions of the dynamic model may not hold.

Ninety percent of reference genomes in WoL are less than 6 million bp in length. By our model, it would take roughly 12,000 reads of length 150 bp to achieve 25% genome cover for a reference of this length. Compositing 100 like samples with roughly 600,000 reads apiece, reaching this threshold requires mean relative abundance 0.02%, in line with standard relative abundance thresholds. This shows that 25% cover is a reasonable threshold for the average microbe to pass in a shallow sequenced 100-sample data set. Table S1 estimates the required number of reads across composited samples to reach a target genome cover threshold within the range of reference lengths in the WoL.

10.1128/msystems.00758-22.2 TABLE S1 Estimated 150 bp reads necessary to achieve specified genome cover thresholds. Download Table S1, DOCX file, 0.01 MB. Copyright © 2022 Hakim et al. 2022 Hakim et al. https://creativecommons.org/licenses/by/4.0/ This content is distributed under the terms of the Creative Commons Attribution 4.0 International license.

Fig. 2a shows that even the weakest cover thresholds filter 80+% of extraneous reference hits in iMSMS. We do not recommend cover thresholds below 10% however, due to the existence of reference species whose genomes highly overlap common members of the gut microbiome. Fig. 2b and c shows that Yersinia pestis (bubonic plague), a false hit that frequently bypasses abundance and prevalence filters (11, 12) has saturated cover around 4.5% in the International Multiple Sclerosis Microbiome Study (iMSMS [13, 14]). This 4.5% cover is due to overlap with Escherichia coli and Klebsiella pneumoniae. Similarly iMSMS covers 9.5% of Bacillus anthracis (*anthrax*) as the result of a few samples containing a close relative of Bacillus thuringiensis (Fig. 2d). As these samples are outliers, it is unclear whether 9.5% is the saturated cover.

**FIG 2:**
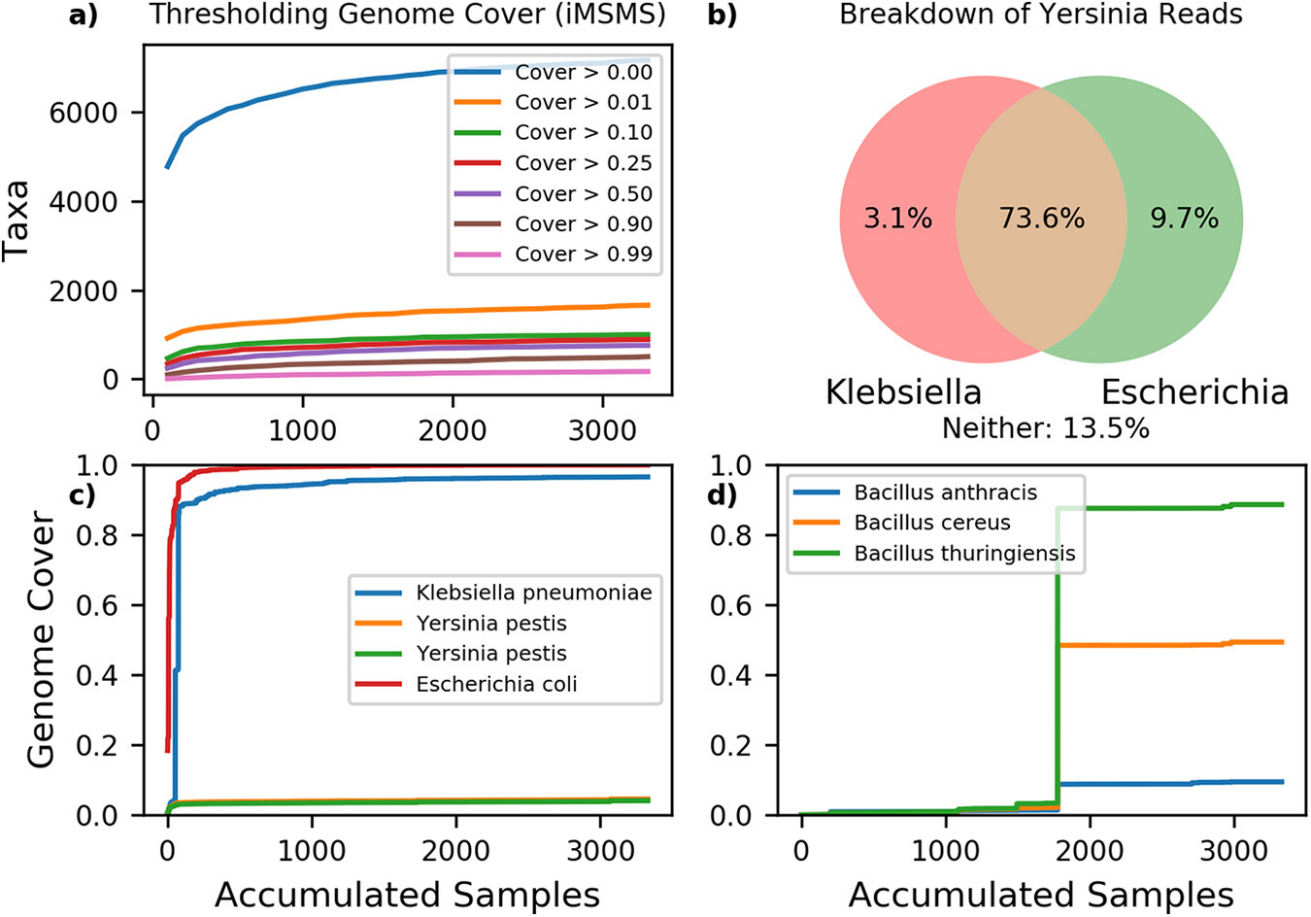
Cumulative genome cover in iMSMS. Genome cover for each reference taxon is accumulated across shuffled iMSMS samples. Sample depth varies from 10^5 to 10^7, median 10^6 reads. (a) Number of taxa passing cover threshold as samples are accumulated. (b) Tracing of reads assigned to Yersinia pestis. (c) Accumulated cover of Yersinia pestis and related microbes by sample. (d) Accumulated cover of Bacillus anthracis and related microbes by sample.

We further caution against cover thresholds above 90%, even with deep sequencing, due to the imperfect nature of a reference. Table 1 shows a 95% cover threshold would remove even S. aureus from the analysis. Table S2 reports genome cover for the WoL relatives of eight ground truth species. Salmonella enterica B4212 only appears to overlap 83% of its nearest relative in WoL. If the reference does not contain close relatives, weaker cover thresholds should be employed.

10.1128/msystems.00758-22.3 TABLE S2 Genome cover of eight member Zymo mock community. Download Table S2, DOCX file, 0.02 MB. Copyright © 2022 Hakim et al. 2022 Hakim et al. https://creativecommons.org/licenses/by/4.0/ This content is distributed under the terms of the Creative Commons Attribution 4.0 International license.

Zebra discards the artifacts of reference overlap, catches problematic species that bypass standard abundance and prevalence filtering, and leads to improved interpretation.
