# Supplementary figures and images for: Zebra: Static and Dynamic Genome Cover Thresholds with Overlapping References
Source: mSystems. 2022 Sep 8;7(5):e00758-22. doi: 10.1128/msystems.00758-22 (PMC9600373; doi:10.1128/msystems.00758-22)

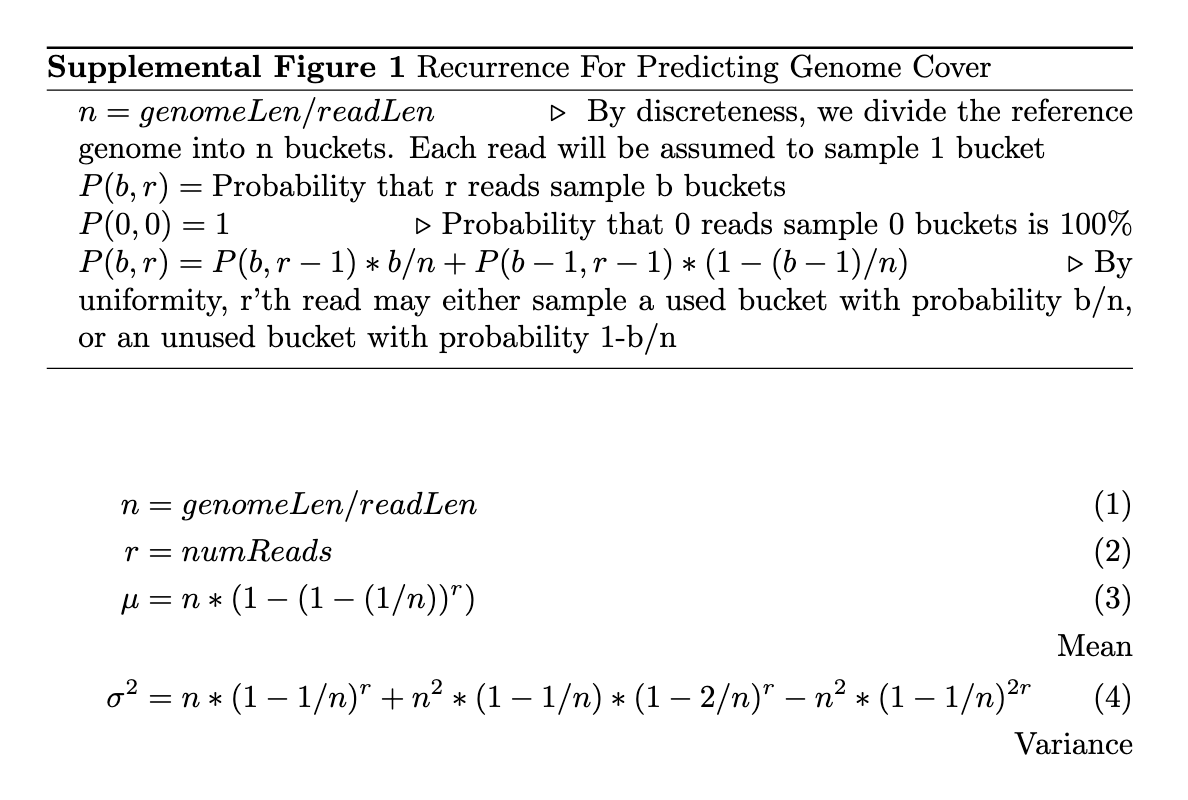

Supplement: Figure S1 [file msystems.00758-22-s0001.tif]
