# Supplementary material for: Zebra: Static and Dynamic Genome Cover Thresholds with Overlapping References
Source: mSystems. 2022 Sep 8;7(5):e00758-22. doi: 10.1128/msystems.00758-22 (PMC9600373; doi:10.1128/msystems.00758-22)
Supplement: TABLE S1 [file msystems.00758-22-s0002.docx]

**Table S1** Estimated 150 bp reads necessary to achieve specified genome cover thresholds

| **Genome Length** | **10% Genome Cover** | **25% Genome Cover** | **50% Genome Cover** | **75% Genome Cover** | **90% Genome Cover** |
| --- | --- | --- | --- | --- | --- |
| 250000 (min) | 180 | 480 | 1200 | 2300 | 3800 |
| 2300000 (25th percentile) | 1600 | 4400 | 11000 | 21000 | 35000 |
| 3400000 (50th percentile) | 2400 | 6500 | 16000 | 31000 | 52000 |
| 4600000  (75th percentile) | 3200 | 8800 | 21000 | 43000 | 71000 |
| 6000000 (90th percentile) | 4200 | 12000 | 28000 | 55000 | 92000 |
| 55000000 (max) | 39000 | 110000 | 250000 | 510000 | 840000 |

Table S1 Low abundance species and species with long reference genomes require more reads to saturate cover, and can fail genome cover thresholds due to lack of reads. These values can be compared with the total number of reads across composited samples to evaluate whether these same species would be discarded by mean relative abundance thresholds
