# Supplementary material for: Zebra: Static and Dynamic Genome Cover Thresholds with Overlapping References
Source: mSystems. 2022 Sep 8;7(5):e00758-22. doi: 10.1128/msystems.00758-22 (PMC9600373; doi:10.1128/msystems.00758-22)
Supplement: TABLE S2 [file msystems.00758-22-s0003.docx]

**Table S2** Genome cover of eight member Zymo mock community

| **OGU** | **Genome Cover** | **WoL Annotation** | **Ground Truth** |
| --- | --- | --- | --- |
| G001283625 | 0.98 | Escherichia coli 401686 | Escherichia coli B-1109 |
| G000472265 | 0.97 | Lactobacillus fermentum Lf1 | Lactobacillus fermentum B-1840 |
| G000159215 | 0.96 | Lactobacillus fermentum ATCC 14931 | Lactobacillus fermentum B-1840 |
| G000740655 | 0.93 | Bacillus sp. BSC154 ASM74065v1 | Bacillus subtilis B-354 |
| G001742205 | 0.92 | Lactobacillus fermentum NCC2970 | Lactobacillus fermentum B-1840 |
| G001297025 | 0.92 | Lactobacillus fermentum HFB3 | Lactobacillus fermentum B-1840 |
| G000648015 | 0.92 | Enterococcus faecalis FL2 | Enterococcus faecalis B-537 |
| G000415085 | 0.91 | Enterococcus faecalis B83616-1 | Enterococcus faecalis B-537 |
| G000619785 | 0.90 | Listeria monocytogenes Lm19860 | Listeria monocytogenes B-33116 |
| G001807655 | 0.90 | Lactobacillus sp. HMSC24D01 ASM180765v1 | Lactobacillus fermentum B-1840 |
| G000397165 | 0.89 | Lactobacillus fermentum F-6 | Lactobacillus fermentum B-1840 |
| G001010245 | 0.88 | Lactobacillus fermentum 90 TC-4 | Lactobacillus fermentum B-1840 |
| G001039735 | 0.88 | Lactobacillus fermentum L930BB | Lactobacillus fermentum B-1840 |
| G001010185 | 0.87 | Lactobacillus fermentum 39 | Lactobacillus fermentum B-1840 |
| G001297905 | 0.87 | Lactobacillus fermentum UCO-979C | Lactobacillus fermentum B-1840 |
| G001456215 | 0.87 | Staphylococcus aureus MS4 | Staphylococcus aureus B-41012 |
| G000648175 | 0.87 | Enterococcus faecalis NJ44 | Enterococcus faecalis B-537 |
| G000010145 | 0.86 | Lactobacillus fermentum IFO 3956 | Lactobacillus fermentum B-1840 |
| G001854105 | 0.84 | Lactobacillus fermentum 47-7 | Lactobacillus fermentum B-1840 |
| G000415005 | 0.84 | Enterococcus faecalis 02-MB-P-10 | Enterococcus faecalis B-537 |
| G000496435 | 0.84 | Lactobacillus fermentum NB-22 | Lactobacillus fermentum B-1840 |
| G000162395 | 0.84 | Lactobacillus fermentum 28-3-CHN | Lactobacillus fermentum B-1840 |
| G000006925 | 0.83 | Shigella flexneri 2a str. 301 | Escherichia coli B-1109 |
| G001368755 | 0.83 | Lactobacillus fermentum 222 | Lactobacillus fermentum B-1840 |
| G000195995 | 0.83 | Salmonella enterica subsp. enterica serovar Typhi str. CT18 | Salmonella enterica B-4212 |
| G000299455 | 0.81 | Escherichia coli O104:H4 str. 2011C-3493 | Escherichia coli B-1109 |
| G001982185 | 0.81 | Lactobacillus fermentum RI-508 | Lactobacillus fermentum B-1840 |
| G001436835 | 0.80 | Lactobacillus fermentum DSM 20055 | Lactobacillus fermentum B-1840 |
| G000012005 | 0.80 | Shigella dysenteriae Sd197 | Escherichia coli B-1109 |
| G000966835 | 0.79 | Lactobacillus fermentum LfQi6 | Lactobacillus fermentum B-1840 |
| G001941785 | 0.79 | Lactobacillus fermentum SNUV175 | Lactobacillus fermentum B-1840 |
| G000026345 | 0.79 | Escherichia coli IAI39 | Escherichia coli B-1109 |
| G000183345 | 0.78 | Escherichia coli O83:H1 str. NRG 857C | Escherichia coli B-1109 |
| G000026325 | 0.78 | Escherichia coli UMN026 | Escherichia coli B-1109 |
| G000007785 | 0.76 | Enterococcus faecalis V583 ASM778v1 | Enterococcus faecalis B-537 |
| G000008865 | 0.76 | Escherichia coli O157:H7 str. Sakai Sakai substr. RIMD 0509952 | Escherichia coli B-1109 |
| G001564695 | 0.73 | Listeria monocytogenes LM06-00983 | Listeria monocytogenes B-33116 |
| G000973005 | 0.72 | Listeria monocytogenes 2KSM | Listeria monocytogenes B-33117 |
| G000196035 | 0.72 | Listeria monocytogenes EGD-e | Listeria monocytogenes B-33118 |
| G001564995 | 0.72 | Listeria monocytogenes LM07-01377 | Listeria monocytogenes B-33119 |
| G001463995 | 0.72 | Listeria monocytogenes 2932 | Listeria monocytogenes B-33120 |
| G000972995 | 0.71 | Listeria monocytogenes 1KSM | Listeria monocytogenes B-33121 |
| G000620885 | 0.71 | Listeria monocytogenes Lm25180 | Listeria monocytogenes B-33122 |
| G000620905 | 0.70 | Listeria monocytogenes Lm21045 | Listeria monocytogenes B-33123 |
| G000567965 | 0.70 | Listeria monocytogenes serotype 1/2a str. 01-1280 | Listeria monocytogenes B-33124 |
| G001663575 | 0.55 | Bacillus subtilis ND23 | Bacillus subtilis B-354 |
| G000696615 | 0.46 | Bacillus subtilis E72 | Bacillus subtilis B-354 |
| G000747645 | 0.46 | Bacillus subtilis Miyagi-4 | Bacillus subtilis B-354 |
| G900110305 | 0.44 | Bacillus subtilis BGSC 3A28 | Bacillus subtilis B-354 |
| G000960735 | 0.28 | Listeria innocua 12KSM | Listeria monocytogenes B-33116 |
| G000183885 | 0.24 | Listeria innocua FSL S4-378 | Listeria monocytogenes B-33116 |
| G000027145 | 0.08 | Listeria seeligeri serovar 1/2b str. SLCC3954 |  |
| G000648515 | 0.06 | Citrobacter freundii CFNIH1 |  |
| G001558935 | 0.06 | Citrobacter amalonaticus FDAARGOS_165 |  |
| G000349845 | 0.05 | Pseudomonas denitrificans ATCC 13867 |  |
| G000689415 | 0.04 | Pseudomonas knackmussii B13 |  |
| G000025565 | 0.04 | Enterobacter cloacae subsp. cloacae ATCC 13047 |  |
| G000982825 | 0.03 | Cronobacter sakazakii ATCC 29544 ASM98282v1 |  |
| G000196735 | 0.03 | Bacillus amyloliquefaciens DSM 7 |  |
| G000694955 | 0.03 | Lelliottia amnigena CHS 78 |  |
| G000164865 | 0.03 | Enterobacter lignolyticus SCF1 |  |
| G001022135 | 0.03 | Kluyvera intermedia CAV1151 |  |
| G001571285 | 0.03 | Kluyvera cryocrescens NBRC 102467 |  |
| G000240185 | 0.03 | Klebsiella pneumoniae subsp. pneumoniae HS11286 |  |
| G000735365 | 0.03 | Kluyvera ascorbata ATCC 33433 |  |
| G000215745 | 0.03 | Enterobacter aerogenes KCTC 2190 |  |
| G001022195 | 0.03 | Klebsiella oxytoca CAV1374 |  |
| G001297765 | 0.03 | Trabulsiella odontotermitis TbO2.3 |  |
| G000300455 | 0.03 | Kosakonia sacchari SP1 ASM30045v4 |  |
| G001941055 | 0.02 | Ruminococcus sp. Zagget7 ASM194105v1 |  |
| G000016325 | 0.02 | Enterobacter sp. 638 ASM1632v1 |  |
| G000735515 | 0.02 | Leclercia adecarboxylata ATCC 23216 = NBRC 102595 ATCC 23216 |  |
| G001517185 | 0.02 | Bacillus velezensis RC218 |  |
| G000247895 | 0.02 | Raoultella ornithinolytica 10-5246 |  |
| G000412695 | 0.02 | Pseudomonas resinovorans NBRC 106553 |  |
| G001617995 | 0.02 | Bacillus amyloliquefaciens H57 |  |
| G001687195 | 0.02 | Bacillus velezensis SRCM100730 |  |
| G000746035 | 0.02 | Bacillus sp. UNC69MF ASM74603v1 |  |
| G000455385 | 0.02 | Pseudomonas alcaligenes OT 69 |  |
| G000235645 | 0.02 | Staphylococcus simiae CCM 7213 |  |
| G900111835 | 0.02 | Pseudomonas otitidis DSM 17224 |  |
| G900156245 | 0.02 | Bacillus amyloliquefaciens GD4a |  |
| G001461825 | 0.02 | Bacillus velezensis NRRL B-41580 |  |
| G001440465 | 0.02 | Bacillus velezensis NBIF-003 |  |
| G000735455 | 0.02 | Yokenella regensburgei ATCC 49455 |  |
| G000275785 | 0.02 | Bacillus sp. 916 ASM27578v1 |  |
| G001597285 | 0.02 | Pseudomonas alcaligenes NEB 585 |  |
| G001461845 | 0.02 | Bacillus velezensis NRRL B-4257 |  |
| G001975995 | 0.02 | Bacillus sp. GZB ASM197599v1 |  |
| G001587325 | 0.02 | Bacillus amyloliquefaciens B4140 |  |
| G000784965 | 0.02 | Pantoea sp. PSNIH2 ASM78496v1 |  |
| G000740715 | 0.02 | Bacillus subtilis NKYL29 |  |
| G001187865 | 0.02 | Klebsiella sp. RIT-PI-d ASM118786v1 |  |
| G000364625 | 0.02 | Pseudomonas thermotolerans DSM 14292 |  |
| G000332735 | 0.02 | Staphylococcus warneri SG1 |  |
| G001471555 | 0.02 | Staphylococcus capitis FDAARGOS_173 |  |
| G000025085 | 0.02 | Staphylococcus lugdunensis HKU09-01 |  |
| G001028645 | 0.02 | Staphylococcus capitis subsp. capitis |  |
| G000262305 | 0.02 | Shimwellia blattae DSM 4481 = NBRC 105725 DSM 4481 |  |
| G000759795 | 0.02 | Escherichia vulneris NBRC 102420 |  |
| G000225325 | 0.02 | Lactobacillus sanfranciscensis TMW 1.1304 |  |
| G000009865 | 0.02 | Staphylococcus haemolyticus JCSC1435 |  |
| G000007645 | 0.02 | Staphylococcus epidermidis ATCC 12228 ASM764v1 |  |
| G000757785 | 0.02 | Pluralibacter gergoviae FB2 |  |
| G000016565 | 0.02 | Pseudomonas mendocina ymp |  |
| G000248015 | 0.02 | Atlantibacter hermannii NBRC 105704 |  |
| G000463155 | 0.02 | Siccibacter turicensis LMG 23730 |  |
| G000425625 | 0.02 | Pseudomonas azotifigens DSM 17556 |  |
| G000757825 | 0.02 | Cedecea neteri SSMD04 |  |
| G000168835 | 0.02 | Yersinia pestis FV-1 |  |
| G000559025 | 0.02 | Luteimonas huabeiensis HB2 |  |
| G000009065 | 0.02 | Yersinia pestis CO92 ASM906v1 |  |
| G000008505 | 0.01 | [Bacillus thuringiensis] serovar konkukian str. 97-27 |  |
| G000621185 | 0.01 | Franconibacter pulveris DSM 19144 |  |
| G000008165 | 0.01 | Bacillus anthracis str. Sterne ASM816v1 |  |
| G000007845 | 0.01 | Bacillus anthracis str. Ames |  |
| G001188915 | 0.01 | Staphylococcus schleiferi 2317-03 |  |
| G000169655 | 0.01 | Yersinia pestis biovar Mediaevalis str. K1973002 |  |
| G000174395 | 0.01 | Enterococcus faecium DO ASM17439v2 |  |

Assigned WoL references are traced back to their most likely source in the known 8 member ground truth. The 8th ground truth microbe, Pseudomonas aerigunosa, does not have a close relative in WoL.
